# Childhood Interstitial Lung Disease—Successful Application of a Stepwise Diagnostic Classification

**DOI:** 10.3390/jcm15103971

**Published:** 2026-05-21

**Authors:** Christina K. Rapp, Matthias Griese

**Affiliations:** Dr. von Haunersches Kinderspital, German Center for Lung Research, University Hospital of Ludwig Maximilian University of Munich, 80337 Munich, Germany; christina.rapp@med.uni-muenchen.de

**Keywords:** chILD, categorization, secondary data analysis, diffuse lung disease, parenchymal lung disease, etiology, non-pulmonary organs, human phenotype ontology, genomics

## Abstract

**Background/Objectives**: Childhood interstitial lung disease (chILD) represents a heterogeneous group of rare pulmonary disorders. Practical diagnostic approaches tested for feasibility and impact in comprehensive cohorts are lacking. We aimed to assess a simple etiologically focused classification approach, clarify the role of genetic testing and quantify the impact of non-pulmonary organ manifestations. **Methods**: We hypothesized that chILD can be classified in a clinically meaningful and versatile way by answering three questions: Which children have an etiological chILD diagnosis due to (1) identified (exposure-related) cause/lung injury, or (2) systemic disease? (3) In how many children without an etiological diagnosis can a genetic cause be identified? We also calculated the predictive value of non-pulmonary organ involvement for underlying systemic conditions. **Results**: Among 1693 patients, 24.7% were grouped as ILD related to exposure, 22.7% as ILD with systemic condition, 16.6% as ILD with genetic diagnosis of systemic disease, 10.0% as ILD with genetic diagnosis affecting the lungs only, and 25.8% as ILD without genetic diagnosis. The average genetic diagnostic yield was 50.8%, with higher rates in interstitial pneumonia (61.4%) or pulmonary alveolar proteinosis (87.1%). The presence of ≥two non-pulmonary organ manifestations increased the likelihood of an underlying systemic disease by three to five-fold. **Conclusions**: An etiological diagnostic strategy effectively classifies chILD and guides genetic testing. Exome or genome sequencing should be considered if ≥two non-pulmonary organs are involved or if the initial diagnosis becomes uncertain due to an unusual disease course or signs of a second underlying condition.

## 1. Introduction

Childhood interstitial lung diseases (chILD), also referred to as pediatric diffuse parenchymal lung diseases (DPLD), represent a wide and diverse spectrum of chronic pulmonary disorders. The currently established classification system stratifies 34 chILD diagnoses, focusing on children younger than two years of age [1], with subsequent expansions incorporating older children [2,3] and non-pathology-based diagnoses [4]. Recent advances in genetic testing often elucidating the precise causes of chILD and generating novel molecularly defined entities [5] challenge current classifications. Prioritizing disease etiologies over phenotypic manifestations [6,7] appears desirable; however, supporting evidence on the successful applicability of such systems is lacking [8]. Subsequent multicenter studies, predominantly retrospective or descriptive, pointed towards the growing role of genetic testing [7,9,10,11,12]. Notably, exome and genome sequencing has broadened genetic interrogation and revealed novel pathogenic variants in previously untargeted genes. Some of these genes had not yet been associated with a disease, and others were not considered because the clinical phenotype was ambiguous. In particular, chILD as part of a systemic condition is increasingly discovered [13,14,15,16,17,18,19]. Nevertheless, the genetic spectrum of chILD remains incompletely mapped. No large study has yet proven the applicability of a comprehensive, practical, and standardized diagnostic classification approach.

Thus, this study employed secondary data analysis of a large cohort of prospectively collected patients with chILD diagnosed by a multidisciplinary team (MDT). We applied a primarily etiological categorization of chILD, which differentiates exposure-related conditions, systemic disease-related disorders and interstitial lung diseases affecting the lungs only [8]. We evaluated each patient by stepwise answering three key questions and classified them accordingly if the ILD was caused by an exposure (Q1), by a systemic condition (Q2), or by a genetic cause (Q3). We tested the hypothesis that this systematic application resulted in a meaningful and versatile classification of a large and heterogeneous cohort of patients with chILD.

We further evaluated the hypothesis that non-pulmonary organ manifestations might serve as important predictors for an underlying systemic disease. To systematically test this, we employed standardized human phenotype ontology (HPO) terminology [20], ensuring consistency in phenotype characterization while facilitating its computational integration and analysis. Together, we quantify the application of an etiological classification system for the first time in a large cohort of patients with chILD and draw conclusions for clinical practice.

## 2. Materials and Methods

### 2.1. Study Design and Participants

The study design is that of a secondary data analysis of prospectively collected cases. This secondary data analysis was conducted at the Department of Pediatric Pneumology, University Hospital Munich, ethically approved by the Ethics committee of the University Hospital Munich (23-0962, date: 25 January 2024), and followed the Declaration of Helsinki and STROBE guidelines [21]. Parents or legal guardians of children participating in the chILD-EU register (www.childeu.net) provided written informed consent for the diagnostic evaluation, processing, storage and usage of their pseudonymized data. For children treated at the pediatric pneumology department in Munich, anonymized data was used as per patient consent and the Ethics committee recommendation.

**Figure 1:**
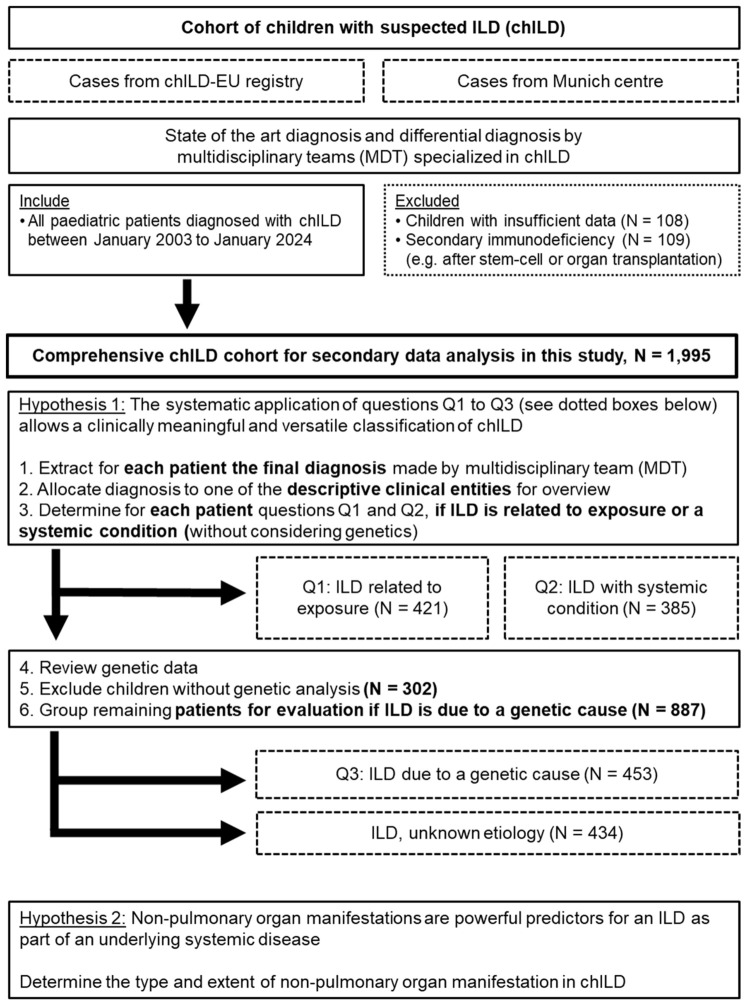
Flow diagram depicting the creation of the chILD cohort investigated, the research hypotheses, and the questions guiding the secondary data analysis.

To ensure a comprehensive and consistent representation of the chILD spectrum, all pediatric patients with suspected chILD who were therefore submitted to the chILD-EU register or the Munich center, between 3 January and 24 January, were investigated. All included patients were evaluated by the same diagnostic work-up, reflecting a standard procedure that has been routinely applied in pediatric pneumology for many years [22] and subjected to the MDT review process. This diagnostic work-up included stepwise and sometimes repetitive testing from less to more invasive methods using clinical history and presentation, high-resolution computer tomography imaging, pulmonary function test, biochemical analyses of blood and broncho-alveolar lavage, genetic testing, and lung histopathology. In patients with suspected pulmonary hypertension, echocardiography or right heart catheterization evaluated a cardiac cause. All test results were reviewed by an MDT of pediatric pneumologists, pediatric radiologists, geneticists and pathologists, all specialized in chILD. Patients diagnosed with chILD were defined as those presenting for longer than 6 weeks with respiratory complaints. These include tachypnea or dyspnea at rest or with exercise, crackles, retractions, predominantly dry cough, hypoxemia (≤92% oxygen), and diffuse radiological abnormalities. To capture the full spectrum of chILD, we intentionally included both children enrolled in the chILD-EU register and those diagnosed outside the register using the same standardized algorithm, creating the broadest possible chILD cohort. Children with insufficient data or secondary immunodeficiency were excluded (Figure 1).

### 2.2. Data Extraction and Secondary Data Analysis

From this cohort, we extracted the final chILD diagnosis made by the MDT, non-pulmonary organ involvement, and the histological diagnosis (if performed) for secondary data analysis in the current study [23]. After careful review, each patient was allocated into a (pre-genetic) clinical entity. We then systematically applied three questions for each patient to achieve an etiology-based classification, using all the extracted data from a patient rather than the clinical entity alone. First, we asked (question Q1) if we can diagnose an identified (exposure-related) cause/lung injury. This included lung prematurity below 30 weeks of gestation and significant respiratory impairment, pulmonary hypoplasia due to lung compression/displacement, and chronic interstitial pneumonia due to infectious (CMV, Mycoplasma, etc.) or non-infectious exposures (IgG-mediated hypersensitivity to antigens, inhaled/systemic drug exposures, and chronic aspiration). All these conditions were classified as “ILD related to exposure”. Next, for each patient, we asked (question Q2) whether systemic disease involvement contributed to the etiology of their ILD. Individuals presenting with a characteristic clinical phenotype consistent with a well-defined condition plausibly accounting for observed ILD manifestations were grouped as having “ILD with systemic condition”. This classification was based on a comprehensive MDT evaluation integrating clinical, imaging, and laboratory findings. Importantly, the MDT decision did not rely on the quantitative number of organ involvement. Organ involvement contributed to but did not determine this classification. ILD with systemic condition included complex syndromic entities, such as collagen vascular disorders, metabolic and autoimmune conditions, immunodeficiencies, cardiovascular abnormalities, and other multi-organ diseases with established associations with ILD. In some cases, children classified as “ILD related to exposure” also exhibited non-pulmonary organ involvement, suggesting the ILD as part of a systemic disease. Those cases were reviewed in detail by the MDT. Any disagreements were resolved through consensus discussion.

All ILDs in patients categorized in response to Q1 or Q2 were considered etiologically solved. All remaining patients underwent a systematic re-evaluation focusing on their previously obtained primary genetic data. Patients without any genetic material or data were excluded. Genetic data were assessed in light of the present knowledge, acknowledging that numerous molecular genetic disorders have been identified since the original testing [13,14,15,16,17,18,19]. The diagnosis, the non-pulmonary organ involvement, histological findings, and the updated genetic results were then used to answer question Q3, determining if an underlying genetic etiology could explain the ILD. If not, the condition was labeled as “ILD with unknown etiology” (Figure 1).

### 2.3. Genetic Testing and Variant Interpretation

Genetic testing evolved over time. At the beginning of the study period, genes frequently associated with ILD were analyzed individually, i.e., *ABCA3*, *SFTPB*, and *SFTPC* (in this study referred to as Sanger sequencing). Later, patients routinely underwent panel-based next-generation sequencing (in this study referred to as panel sequencing). Common genes, including *ABCA3*, *CSF2RA*, *CSF2RB*, *FLNA*, *SFTPB*, *SFTPC*, *MARS1*, *NKX2-1*, and *FOXF1*, were analyzed and complemented by *ACVRL1*, *BMPR2*, *EIF2AK4*, *ENG1*, *SMAD4*, and *TBX4*, if pulmonary hypertension was suspected. Since 2019, exome sequencing has been performed more routinely, including re-analysis of previously unsolved cases. All genetic variants were interpreted according to the current state of knowledge and consistently categorized using ACMG guidelines [24]. Variants detected and classified as (likely) pathogenic in clinical databases (e.g., ClinVar) were re-evaluated for their supporting evidence. If they were appropriate and consistent with the patient’s phenotype, the genetic diagnosis was considered identified. For novel variants, it was essential that the type of consequence, the genotype and observed frequency in control databases (gnomAD) were consistent with linked human disease (OMIM). All identified genetic diagnoses were proven by literature research, if they were previously associated with ILD or other conditions which can cause secondary ILD. If parental material was available, variants were verified to segregate with the disease. An exception was made for genes with known incomplete penetrance (e.g., *BMPR2* [25] and *COPA* [26]), in which an asymptomatic parent may carry the variant without exhibiting clinical signs.

### 2.4. Evaluation of Non-Pulmonary Organ Manifestation

The clinical presentation, particularly non-pulmonary organ involvement, was coded by standardized HPO terms [20]. All terms were linked to major organ groups: (1) autoimmune, (2) immunological, (3) hematological, (4) cardiac, (5) vascular, (6) lymphatic, (7) gastrointestinal (including pancreatic), (8) hepatic (including gall bladder), (9) nephrotic (including the urogenital system), (10) skeletal, (11) muscular, (12) neurological, (13) dermal (including hair/nails), (14) endocrine, and (15) syndromic (including head/neck) abnormalities. From these, data frequencies of organ involvement and probabilities for the presence of a systemic disease were calculated.

### 2.5. Statistical Analysis

Statistical analyses were performed using Prism v.9.3.1. The cohort characteristics are presented as median ± IQR or percentage of each cohort. Differences were assessed by one-way ANOVA with Tukey post hoc testing, with statistical significance defined as *p* < 0.05. The average number of HPO terms and involved organs was given as mean ± SD. Simple and multiple logistic regressions were performed with systemic disease present or absent as the dependent variable, and as predictors, the number of non-pulmonary organs and the type of organ involved were used. No additional covariates were included. The results were indicated as odds ratios with 95%CI (profile likelihood). A 95%CI different from 1 was considered statistically significant; no correction for multiple testing was applied. To independently quantify the association between organ involvement and systemic disease, we constructed a 2 × 2 contingency table and calculated statistical significance using a chi-square test and estimated the effect size with the corresponding odds ratio with 95% confidence intervals.

## 3. Results

### 3.1. Patients’ Characteristics and Allocation into Pre-Genetic Clinical Entities

**Figure 2:**
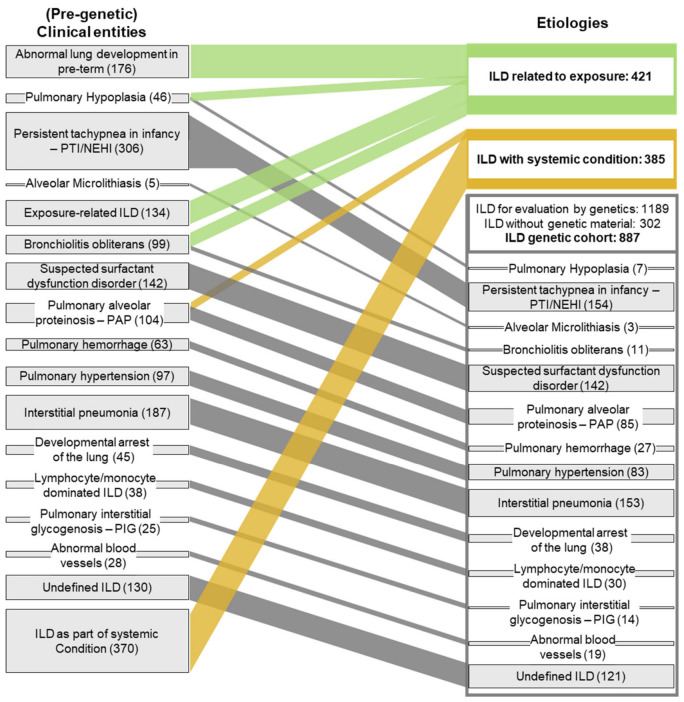
Overview of (pre-genetic) clinical entities and grouping of subjects into etiologic entities. The 1995 children were first allocated into (pre-genetic) clinical entities, reflecting a descriptive, non-genetic, commonly used catalog of the chILD-EU register (left column). Then, patients were grouped based on the etiology of their conditions (right column). This was systematically done by answering questions 1 and 2, i.e., if an exposure-related cause (green connectors) or a systemic disease connected to their ILD (orange connectors) fully explained their ILD. After the exclusion of children with no genetic analysis (see Figure 1), the remaining children built the cohort for evaluation by genetics (the ILD genetic cohort (gray connectors)).

The database for this study was extracted from a large collection of patients with chILD with state of the art’s diagnosis and differential diagnosis conducted by an MDT. In total, 1995 children with a confirmed diagnosis of ILD were included. Their median age at baseline assessment was 1.3 years, and there were more males than females (ratio 1:0.82), with 56.1% being below the age of 2 years. The follow-up time was 7.1 (IQR 3.0–13.2) years, and 19.2% died or were lung transplanted at a median age of 0.5 (IQR 0.1–3.4) years. The characteristics of the two cohorts are summarized in Table S1. All children underwent an individual review of their final MDT-established chILD diagnosis, non-pulmonary organ involvement, and histology. Based on this information, they could be easily and unconstrainedly allocated into one of 17 (pre-genetic) clinical entities, reflecting a simplified disease catalog of the chILD-EU register. These entities were broadly defined, ordered with increasing complexity of their diagnostic procedures and structured the huge number of different chILD diagnoses in a commonly accepted descriptive manner (Figure 2, left column).

### 3.2. Application of a Simple Etiological Classification Approach for chILD

We used each patient’s MDT diagnosis and data to investigate the applicability of a simple classification approach for chILD, answering three consecutive questions, similar to those in everyday clinical practice:

1. In which children can we make an etiological diagnosis due to an identified cause/lung injury (“ILD related to exposure”)?

In 421 children, we recognized an identified exposure-related cause or lung injury (Table 1, Figure 2). These children came from four clinical entities, i.e., abnormal lung development due to extreme prematurity, pulmonary hypoplasia, exposure-related ILD and post-infectious bronchiolitis obliterans, with their relative frequencies listed in Table 1.

2. In which children can we make the etiological diagnosis of a specific ILD as part of a systemic disease “ILD with systemic condition”?

In 385 children, the respiratory symptoms were most likely attributable to an underlying systemic condition, often diagnosed prior to pulmonary manifestations (Table 1, Figure 2). We attributed all symptoms to a single condition, acknowledging the impossibility to distinguish if symptoms arose from a systemic disease or occurred independently, as two diseases. The systemic conditions and their frequencies are listed in Table 1. Application of these two questions was able to etiologically recognize 806 of the 1995 children (Figure 2). Among the remaining 1189 children, no disease etiology was identified, prompting an evaluation of the role of genetic testing; however, 302 children lacked genetic material or data and thereby could not be analyzed further.

3. In how many children without an etiological ILD diagnosis based on an exposure or a systemic condition can we identify a genetic cause and which?

Of the remaining 887 children who underwent genetic testing as part of their ILD work-up, more than half (453 of 887; 51.1%, Table 2) received a causal genetic diagnosis. In children with pulmonary alveolar proteinosis, suspected surfactant dysfunction disorders (low SP-B or SP-C in lavage) or interstitial pneumonia causal variants were identified in 87.1%, 70.2%, and 61.4%. In about two-thirds of the patients with suspected surfactant dysfunction disorder and half of the patients with interstitial pneumonia, a disease-causing variant in *ABCA3*, *SFTPC*, or *SFTPB* was identified. These genetic etiologies, along with patients harboring disease-causing variants in *SLC34A2*, were collectively grouped as having “ILD with genetic diagnosis affecting the lungs only”. In total, this subgroup comprised 170 patients (19.2%, 170 of 887). Beyond those known candidate genes for ILD, in 283 patients (31.9%, 283 of 887), 89 different genetic diagnoses associated with systemic diseases were identified (Table 2). These patients were grouped as having “ILD with genetic diagnosis of systemic disease”.

**Figure 3:**
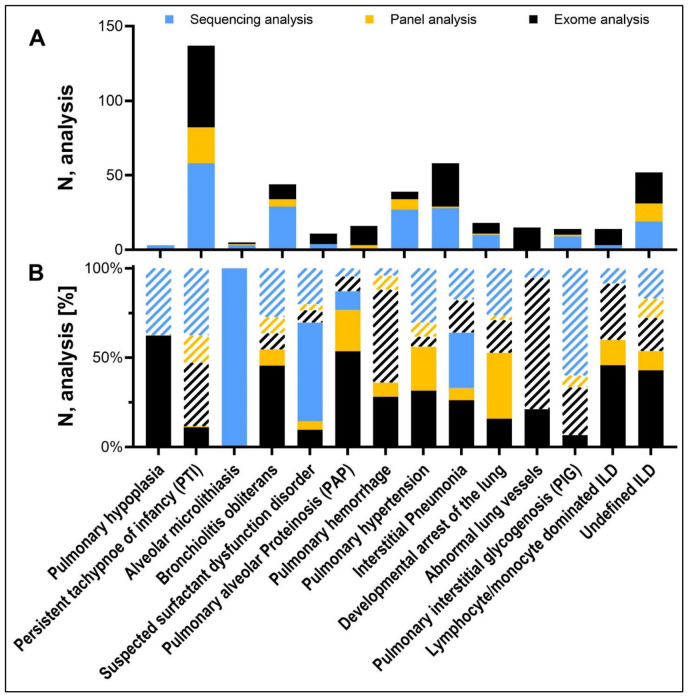
(**A**) Different types of genetic testing per individual clinical entity in total numbers in patients with ILD of unknown etiology (N = 887) and (**B**) their success rate, showing if genetic etiology was identified (N = 453) or not (N = 434; white stripes). Sanger in blue, panel sequencing in yellow and exome sequencing in black.

In 434 patients, no causal genetic variant could be identified (Figure 3). These patients predominantly originated from clinical entities for which either no genetic cause has yet been established or where a monogenetic etiology is considered unlikely. These conditions represented the lowest diagnostic yield at 7.1% and 0%, respectively (Table 2).

### 3.3. Occasional Genetic Testing Among Children with an ILD Related to Exposure or ILD with a Clinical Diagnosis of Systemic Disease

Notably, among 806 patients with an etiological chILD diagnosis, i.e., those two groups generated in response to questions Q1 and Q2, genetic testing was occasionally performed. This was usually requested by the attending physician to confirm the diagnosis, formally exclude alternative conditions, or refine differential diagnoses (Table 3). In the group of 421 children with “ILD related to exposure”, genetic testing was performed in 151 cases. Among these, eight children (5.3%) were unexpectedly found to have a systemic condition. Some of these conditions might be related to the pulmonary phenotype observed, such as recurrent aspiration pneumonia in four patients with underlying syndromic disease and PIBO in a patient with immunodeficiency. Others were likely unrelated, including abnormal lung development in two preterm infants with underlying syndromic disease and pulmonary hypoplasia in an infant with osteogenesis imperfecta type XI (Table 3). Importantly, none of these children had genetic variants in well-known childhood ILDs, such as surfactant dysfunction disorders. In the next group of children with “ILD with systemic condition”, genetic testing was performed in 216 cases, which successfully excluded surfactant dysfunction disorders or confirmed the systemic condition in 98.6% (Table 3). However, in three cases, pathogenic variants were incidentally and unexpectedly identified, in which the causal gene does not explain the observed ILD. One patient had an autoimmune PAP and genetically identified common variable immunodeficiency, and two had Lane Hamilton syndrome and an underlying syndrome or type-I-interferonopathy (Table 3).

### 3.4. Non-Pulmonary Organ Involvement Increases the Likelihood of an Underlying Systemic Disease Etiology for ILD by Three to Five-Fold

**Figure 4:**
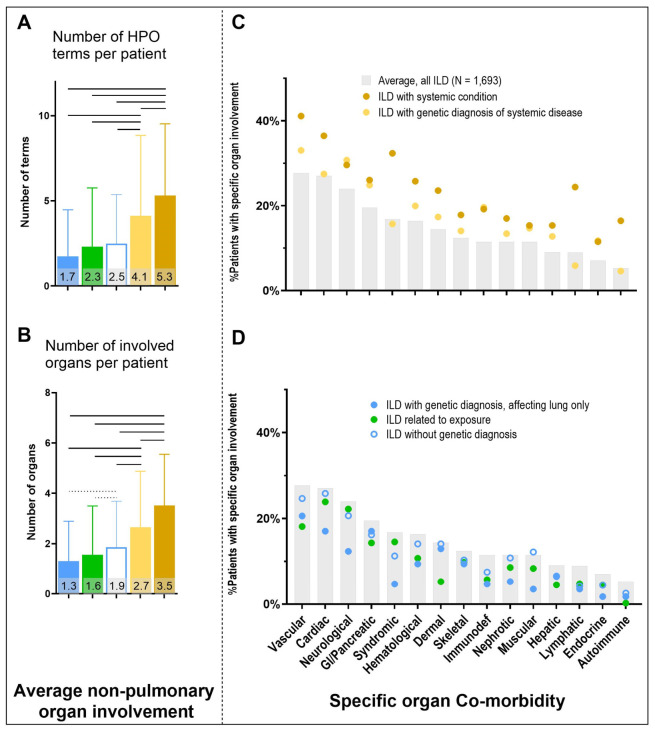
Non-pulmonary organ involvement in the five etiological cohorts: ILD with genetic diagnosis affecting the lungs only (blue), ILD related to exposure (green), ILD without genetic diagnosis (blue outline), ILD with genetic diagnosis of systemic disease (bright yellow), or ILD with convincing diagnosis of systemic disease (dark yellow). (**A**): average number of HPO terms per patient describing non-pulmonary organ involvement and (**B**): the average number of individual organs involved per patient (Data are given as mean ± SD; significance is depicted as solid line: *p* < 0.0005 and dotted line: *p* < 0.05). (**C**,**D**): Differentiation of organ involvement into the organ-specific co-morbidity. All patients (N = 1693), gray columns, patients with underlying systemic diseases and patients with ILD related to exposure, ILD with genetic diagnosis affecting the lung only and ILD without genetic diagnosis.

We systematically recorded the non-pulmonary organ involvement in all 1693 patients with chILD to evaluate their role in the etiological diagnosis of ILD. Patients with ILD as part of a systemic condition (genetic or clinical diagnosis) had approximately threefold more HPO terms and two-fold more organs involved (Figure 4A,B). Children with ILD related to exposure had, on average, 2.3 organs involved. Retrospectively, 167 of 421 individuals exhibited involvement of two or more organs, most commonly observed in individuals with pulmonary hypoplasia from external compression (n = 24; 69%), BPD (n = 95; 57%), aspiration pneumonia (n = 13; 33%), and PIBO (N = 19; 25%).

**Table 3:** Results of occasional genetic testing among children with an ILD related to exposure (in response to question Q1, N = 421) or ILD with a clinical diagnosis of systemic disease (in response to Q2, N = 385), allowing an estimate of classification error. Incidental genetic findings consisted of two distinct categories: * genes that were related to the ILD phenotype, suggesting that the patient may have been misclassified; ** disease-causing genes that do not explain the observed ILD phenotype but were identified incidentally.

Clinical Entities	Final Diagnosis	All	Genetically Analyzed	Exclusion of ILD, Only or Confirmation of Systemic Condition	Incidental Finding	Gene Name of Incidental Findings
**ILD related to exposure**		**421**	**151 (35.9%)**	**143 (94.7%)**	**8**	
Abnormal lung development in pre-term	Bronchopulmonary dysplasia in preterm, BPD-cLDI (≤30 weeks of gestation)	176	89 (50.7%)	87 (97.8%)	2	6q23 del ** distal trisomy 16p **
Pulmonary hypoplasia	Pulmonary hypoplasia from external compression	35	15 (42.9%)	14 (93.3%)	1	FKBP10 **
Exposure-related ILD	Aspiration syndrome (42), infectious agent induced chronic ILD (15), inhalation exposure (61), lung dysmaturation from intrauterine exposure alcohol, drugs, diabetes (5), related to radiation/drugs/toxins (11)	134	28 (35.8%)	24 (85.7%)	4	22q11.2del *, 2q15del *, Monosomy 21 *, 5q31.3 del *
Bronchiolitis obliterans	Infectious/post-infectious processes + BO (PIBO)	76	19 (25%)	18 (94.7%)	1	IFIH1 *
**ILD with systemic condition**		**385**	**216 (56.1%)**	**213 (98.6%)**	**3**	
Pulmonary alveolar Proteinosis, PAP	Autoimmune PAP	15	7 (46.7%)	6 (85.7%)	1	TNFRSF13B **
ILD as part of systemic condition	Antibody deficiency (9), antisynthetase syndrome (3), autoinflammatory disorder (4), chronic autoimmune disorder (9), interferonopathy (9), other immune dysregulation disorders (8), phagocyte deficiency (2), (severe) combined immunodeficiency (7), syndromic immunodeficiency (6), Birt-Hogg-Dube syndrome (1), collagen vascular disorders/mixed connective tissue disease (19), congenital musculoskeletal disorder (5), Ehlers-Danlos (2), Hermansky–Pudlak syndrome (10), storage disorders (8), Lysinuric proteinuria (7), Marfan syndrome (3), neurological disorder (13), Niemann–Pick disease (9), Noonan syndrome (8), eosinophilic granulomatosis with polyangiitis—EGPA (9), granulomatosis with polyangiitis—GPA (17), Langerhans cell histiocytosis (7), sarcoidosis (19), Down syndrome (59), Klinefelter syndrome (3), systemic disease with/without dysmorphic features (32), systemic lupus erythematosus (6), systemic sclerosis (3), hereditary hemorrhagic telangiectasia/M. Osler (8), congenital heart disease/cardiac dysfunction (33), vessel disease (32)	370	209 (57.0%)	207 (99.0%)	2	CDK13 **, COPA **
		**806**	**367 (45.1%)**	**356 (97.0%)**	**11**	

Overall organ involvement was then grouped into organ-specific comorbidities and ranked by their occurrence, showing vascular, cardiac, neurological, and gastrointestinal organs as most commonly affected in all children with ILD (Figure 4C,D). ILD with clinical diagnosis of systemic disease, and to a slightly lesser extent, patients with genetic diagnosis of systemic disease, demonstrated more frequent involvement across all organs (Figure 4C).

**Figure 5:**
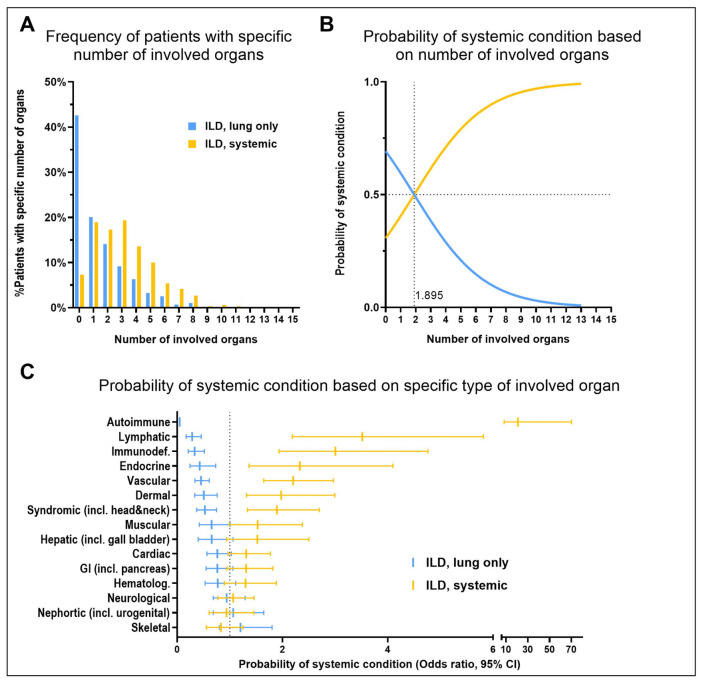
Probability of an underlying systemic disease in children with ILD. (**A**) Frequency of patients with one to fifteen involved organs among patients with an isolated lung disease (in blue: patients with identified exposure and patients with genetic diagnosis of ILD; “ILD, lung only”) compared to patients with ILD as part of an underlying systemic condition (in yellow: clinical or genetic diagnosis of systemic disease; “ILD, systemic”). (**B**) Probability of an underlying systemic condition based on the number of affected organs. (**C**) Results of multiple logistic regression, given the observed probability of a systemic condition depending on the type of affected organs; data is given as odds ratio with 95% CI.

To evaluate whether the extent of non-pulmonary organ involvement predicts the presence of a systemic disease, we performed a logistic regression analysis including all patients with an identified etiology (n = 1259). Patients were stratified into those with ILD as part of a systemic condition (‘ILD, systemic’), defined either clinically or genetically, and those with ILD as an isolated lung condition (‘ILD, lung only’), attributed to an exposure or a genetic diagnosis affecting the lungs only. Notably, over 65% of patients grouped as ‘ILD, lung only’ had no or only one additional organ involvement (Figure 5A). When two or more organs were affected in a patient with ILD, the likelihood of an underlying systemic disease surpassed 50%, further increasing as the number of involved organs rose (Figure 5B). In the corresponding 2 × 2 analysis, the association between ≥2 organ involvement and systemic disease was highly significant (*p* = 2.6 × 10^−10^), with an effect size of OR 4.72 (95% CI 3.71–5.99).

Given the non-negligible frequency of non-pulmonary organ involvement in general (Table S2), the impact of specific organ systems was assessed. The presence of autoimmune, lymphatic, immunodeficiency, endocrine, vascular, dermal, or syndromic manifestations was associated with a significantly increased probability of an underlying systemic condition (Figure 5C, 95%CI > 1). In contrast, neurological, nephrotic, and skeletal abnormalities were prevalent in both subgroups with no predictive differentiation.

## 4. Discussion

This study applied a practical, stepwise diagnostic classification to a large cohort of 1995 patients with chILD, all previously diagnosed by an MTD. The approach enabled clear etiological grouping based on simple, well-defined criteria and demonstrated several advantages.

### 4.1. Etiology-Based Grouping Can Help Guide Patient Care

Etiology-based grouping supports clinical decision-making. In children with an etiological diagnosis due to an identified exposure-related cause, eliminating ongoing exposures is critical. A wait-and-see approach may be appropriate in cases of past lung injuries resulting from lung immaturity or displacement/compression. If disease progression is unexpectedly severe or recurrent infections suggest an underlying immunologic disorder, exome or genome sequencing should be reconsidered. The same applies when recurrent aspiration events raise concern for a neurological or syndromic condition. In children with an ILD due to a systemic disease, multidisciplinary collaboration involving non-pulmonary subspecialties may be required for comprehensive patient management. Identifying systemic diseases underlying chILD enhances prognostic accuracy and enables rational selection of targeted, personalized treatment strategies and monitoring for disease-related complications before they occur. Even as genetic testing becomes more accessible and affordable, we believe that broad genetic testing is not justified in all suspected chILD cases. Despite its benefits, advanced genetic testing presents challenges, particularly the need for competent genetic consulting to ensure patients and families receive accurate interpretation, informed decision-making, and appropriate clinical guidance [27,28]. Lastly, the resource-intensive nature, including time for interpretation and data storage, needs to be considered.

### 4.2. High Yield of Etiology-Based Genetic Diagnostics in chILD

In a well-defined cohort of etiologically undiagnosed chILD, a causal genetic diagnosis was achieved in 51% of cases (453 of 887). Within specific clinical entities such as suspected surfactant dysfunction disorders or pulmonary alveolar proteinosis, the diagnostic yield ranged from 70% to 87%, largely reflecting variants in established ILD-associated genes. In contrast, entities such as interstitial pneumonia or undefined ILD rely predominantly on broad genetic testing, as targeted approaches would miss a substantial proportion of diagnoses. This is consistent with findings of other studies using targeted sequencing alone, which reported lower diagnostic yields [29]. Importantly, even when genetic analysis is inconclusive, exome data allows for future reinterpretation as genomic knowledge evolves, underscoring the long-term value of early integration of genetic testing into clinical workflows. This is especially important, given the broad genetic heterogeneity of ILD with a continuously expanding set of validated gene–disease relationships [30]. Our data supports the view that genetic testing may reduce the need for lung biopsies. The number of lung biopsies declined steadily over 5-year periods, particularly after the widespread adoption of exome sequencing in 2019: from 81 cases in 2010–2014, to 79 in 2015–2019, and only 49 in 2020–2024. Simultaneously, the number of genetic tests performed increased from 159 to 353 to 439, respectively.

### 4.3. Flexible Adaptation to Diagnostic Advances

Not linking a classification system to specific diagnostic tests, such as lung biopsies [1], avoids creating rigid hierarchies and allows for the seamless integration of newly discovered diseases and emerging genomic insights under common clinical entities. This flexibility is critical in a rapidly evolving diagnostic landscape. In our cohort, genetic analysis led to the reclassification of a substantial subset of patients by the identification of a genetic diagnosis in 453 of 1693 children. Among these, 283 (16.6%) were reassigned from morphology-based or unclassified ILD categories to “ILD with genetic diagnosis of systemic disease”. This expanded the currently established diagnostic catalog with 34 entities by 89 systemic genetic diagnoses. Some of those conditions (e.g., *COPA*, *TMEM173*, *and MARS1*) were suspected but not diagnosed clinically and subsequently confirmed by genetic testing. Other conditions were newly identified through the detection of 28 pathogenic variants in 23 genes not previously linked to an ILD phenotype (Table 2, marked with an asterisk). We conclude that broad genetic testing facilitated the discovery of potential novel candidate genes.

### 4.4. Role of Non-Pulmonary Organ Involvement in Patient Management

In a large chILD cohort, we determined the prevalence of 179 distinct etiological chILD diagnoses (Table 2 and Table 3), of which approximately 40% were systemic conditions. We also quantified for the first time the extent of non-pulmonary organ involvement in chILD, demonstrating that the presence of ≥two non-pulmonary organ manifestations increased the likelihood of an underlying systemic disease by three to five-fold. Remarkably, approximately 17% (47 of 283) of patients with a genetic diagnosis of systemic disease exhibited no additional organ manifestation. This finding is clinically significant, as it suggests that certain characteristic symptoms may be subtle, overlooked, or emerge later during the disease course.

Non-pulmonary organ involvement was frequently observed even in children without an underlying systemic condition, particularly in exposure-related ILD. Among these, comorbidities were most commonly observed in the same clinical entities, as occasional genetic testing uncovered a small number of previously unrecognized systemic conditions. Although the detection rate was low, these findings indicate that marked multisystem involvement can point toward an underlying systemic disorder, even when the initial clinical impression suggests an external injury or isolated pulmonary process. At the same time, the overall error rate remained minimal, and surfactant-related disorders were never identified in this context, supporting a selective rather than routine use of genetic diagnostics when ILD is clearly attributable to an exposure. Thus, while extensive organ involvement alone should be carefully interpreted, it may serve as a useful prompt to consider broader genetic testing when the phenotype does not fully align with the presumed diagnosis.

Beyond the number of affected organs, the type of organ affected, particular when immune-, endocrine-, vascular-, dermal-systems are involved or syndromic features are present, should raise suspicion for systemic diseases. 

Consequently, and in summary, exome or genome sequencing may be considered if ≥ two non-pulmonary organs are involved, the initial diagnosis made does not fit the expected disease course or a second underlying condition is suspected.

### 4.5. Limitations

Some aspects of this study may limit the interpretation of the findings. The selection of subjects with chILD may be biased by the cases investigated. We tried to reduce this risk by including a very large number of patients who had undergone the MDT review process. We also increased the representativeness of the cohort by including the Munich cohort. This likely compensated for a potential inclusion of rarer or more complex cases coming from the registry. Although we believe that the results are generalizable, this needs to be confirmed in future validation studies, using different cohorts.

Not all patients received the same genetic testing approach, as many were evaluated with targeted sequencing only. Among 887 genetically analyzed patients, 234 underwent targeted sequencing alone, which remained inconclusive. In these cases, exome sequencing may help close the diagnostic gap and identify additional genetic diagnoses. To account for the 302 children without genetic material/results who were excluded from the final analysis, we reported a clinical entity-specific adjusted diagnostic yield.

This study also reported several previously undescribed variants in genes with an as-yet-unproven link to ILD. Although the recurrence of variants in several unrelated individuals suggested a possible connection, further data are necessary to prove this relation. These may come from functional studies, which are beyond the scope of this paper [31], or represent a starting point for others to identify similar cases.

The role of non-pulmonary organ involvement may be refined by a more detailed consideration of the HPO terms, e.g., by taking into consideration the temporal development of symptoms or the relative impact of certain terms above others. In this line, more detailed analyses in specific disease subgroups may provide further insights.

## 5. Conclusions

Here, we show for the first time in chILD that a stepwise, diagnostic classification approach, focusing on the disease etiology, can be successfully applied. By allocating patients in response to the answers to three questions regarding disease etiology, this study shows that a clear, versatile and clinically relevant grouping of a large number of chILD conditions is feasible.

Integrating advanced genetic testing substantially increased the diagnostic yield, may reduce the number of invasive procedures, and enable earlier recognition of systemic diseases, even when extra-pulmonary signs are subtle or absent. The flexible classification framework adapts well to ongoing genomic advances and supports the discovery of new gene–disease relationships.

Lastly, our data quantitate for the first time in chILD the value of considering non-pulmonary organ involvement. The presence of ≥two organ systems in addition to lung involvement in a patient should alert for the search of an underlying systemic condition, of which the lung disease may be part of.

Taking the etiology-focused stepwise approach into clinical practice may help classify the broad spectrum of chILD in a sustainable way and guide genetic testing.

## Figures and Tables

**Table 1:** Clinical entity and final diagnosis of the 806 children grouped based on their etiology by answering questions 1 and 2, if an exposure-related cause or clinical diagnosis of systemic diseases could be identified in children with ILD.

Clinical Entities	Etiological Diagnosis	N
**ILD related to exposure**		**421**
Abnormal lung development in pre-term	Bronchopulmonary dysplasia in preterm—BPD-cLDI (≤30 weeks of gestation)	176
Pulmonary hypoplasia	Pulmonary hypoplasia from external compression	35
Exposure-related ILD	Aspiration syndrome (42), infectious agent-induced chronic ILD (15), inhalation exposure (61), lung dysmaturation from intrauterine exposure alcohol, drugs, diabetes (5), related to radiation/drugs/toxins (11)	134
Bronchiolitis obliterans	Post-infectious bronchiolitis obliterans (PIBO)	76
**ILD with systemic condition**		**385**
Pulmonary alveolar proteinosis, PAP	Autoimmune pulmonary alveolar proteinosis—aPAP	15
ILD as part of systemic condition	Antibody deficiency (9), antisynthetase syndrome (3), autoinflammatory disorder (4), chronic autoimmune disorder (9), interferonopathy (10), other immune dysregulation disorders (8), phagocyte deficiency (2), (severe) combined immunodeficiency (7), syndromic immunodeficiency (6), Birt–Hogg–Dube syndrome (1), collagen vascular disorders/mixed connective tissue disease (19), congenital musculoskeletal disorder (5), Ehlers–Danlos (2), Hermansky–Pudlak syndrome (10), storage disorders (8), Lysinuric proteinuria (7), Marfan syndrome (3), neurological disorder (13), Niemann–Pick disease (9), Noonan syndrome (8), eosinophilic granulomatosis with polyangiitis—EGPA (9), granulomatosis with polyangiitis—GPA (17), Langerhans cell histiocytosis (7), sarcoidosis (19), Down syndrome (59), Klinefelter syndrome (3), systemic disease with/without dysmorphic features (32), systemic lupus erythematosus (6), systemic sclerosis (3), hereditary hemorrhagic telangiectasia/M. Osler (8), congenital heart disease/cardiac dysfunction (33), vessel disease (32)	370
		**806**

**Table 2:** Genetic investigation of ILD genetic cohort, N = 887. Patients were grouped by their clinical entity and genetic etiologies identified (for the overview, only the causal genetic identifier is given under etiological diagnosis). The frequency of individuals (n) in which a given gene was detected is given in brackets if identified more than once; if detected once, only the name of the gene is indicated. In addition to the number of genetically analyzed patients and the diagnostic yield, the number of patients without available genetic material is also indicated, allowing to calculate a conservative adjusted diagnostic yield.

Clinical Entities	Etiological Diagnosis (N)	Genetically Analyzed	Diagnostic Yield	No Genetic Material	Adjusted Diagnostic Yield	All
Pulmonary hypoplasia	EYA1 *, FGF10, TBX4, THOC6	7	57.1%	4	36.4%	11
Persistent tachypnea of infancy, PTI/NEHI	NAA10 * (3), SRRM2 (4), BRWD3 *, DEPDC5 *, NKX2-1, UBE3B	154	7.1%	152	3.6%	306
Alveolar microlithiasis, PAM	SLC34A2 (3)	3	100.0%	2	60.0%	5
Bronchiolitis obliterans	AGR2 (2), CAV1 *, CYBA, DOCK8, MCM4	11	54.5%	12	26.1%	23
Suspected surfactant dysfunction disorder	ABCA3 (53), SFTPC (28), SFTPB (19), SFTPA1	142	70.4%		70.4%	142
Pulmonary alveolar Proteinosis, PAP	MARS (37), CSF2RA (19), SFTPC (8), ABCA3 (2), OAS1 (2), CSF2RB, FARSA, IARS1, PNP, SFTPB, TBX4	85	87.1%	4	83.1%	89
Pulmonary hemorrhage	COPA (4), TBX4 (3), FGF10, NLRP3	27	33.3%	36	14.3%	63
Pulmonary hypertension	TBX4 (12), FLNA (10), FOXF1 (2), BMPR2 (2), ENG (2), 11q23 del *, 15qdel *, 15q11dup *, 19q13dup *, 7q11del *, ACTA2, COL4A1, CDK13, COQ2, EIF2AK4, KCNK4 *, G6PD *, PIEZO1, SLC25A26, TMEM173	83	53.0%	14	45.4%	97
Interstitial Pneumonia	ABCA3 (38), SFTPC (14), SFTPB (3), FARSB (4), FARSA (3), COPA (3), IFIH1 * (3), NKX2-1 (3), CCR2 (2), STAT3 (2), FLNA (2), ZNFX1 (2), TMEM173 (2), ATM, CD40 *, CYBA, GATA2, MEFV, NCF2, NHLRC2, NLRP3, PEX1, PLCG2, TBX4, TERC, TERT	153	61.4%	34	50.3%	187
Developmental arrest of the lung	FOXF1 (16), STRA6 (2), FGF10,	38	50.0%	7	42.2%	45
Lymphocyte/monocyte dominated ILD	COPA (2), STAT3 (2), COG7 *, CYBA, CYBB, IL2RG, IRF2BP2 *, LRBA, NKX2-1, SCNN1A, SFTPC, SYNE1, TERT, TNFRSF13B, WFS1 *	30	56.7%	8	44.7%	38
Pulmonary interstitial glycogenosis, PIG	-	14	0.0%	11	0.0%	25
Abnormal lung vessels	CAPNS1, EIF2KA, KDM3B, WNT5A	19	21.1%	9	14.3%	28
Undefined ILD	NKX2-1 (14), TMEM173 (13), FLNA (3), CYBB (2), DKC1 (2), FARSB (2), FOXF1 (2), STAT3 (2), TERT (2), ZNFX1 (2), 5q trisomy *, 9p Monosomy *, Xp22.33 dup *, ACTA2, ADA, ATM, ATR *, CDK13, CSF2RA, DOCK8, FOXP3, HBA2, HBB, IFIH1 *, IL2RG, KDM6A *, NHLRC2, PCDH19 *, PLCG2, RASA1, RTEL1, SLC25A20, TBX4, TOP2B, TSC1	121	56.2%	9	52.3%	130
	**453**	**887**	**51.1%**	**302**	**38.1%**	**1189**

* Incidental findings refer to variants that do not explain the ILD phenotype of the patient based on current knowledge.

## Data Availability

Anonymized aggregated data from the chILD-EU register are available upon reasonable request. Research proposals for such data can be submitted to the corresponding author (matthias.griese@med.uni-muenchen.de) and will be assessed by the chILD-EU register coordination team. Aggregated data will be shared if the proposal addresses relevant research questions. Requests for anonymized individual patient data should also be directed to the corresponding author, who will facilitate contact with the responsible physicians to discuss the legal and administrative feasibility of data sharing. Preprint 10.1101/2025.09.14.25335703 was published on Medrxiv.

## Supplementary Materials

The following supporting information can be downloaded at: https://www.mdpi.com/article/10.3390/jcm15103971/s1, Table S1: Demographic data of patients with chILD included in secondary data analysis. Data were given as total number and percentages per group or median with first and third quartiles; Table S2: Most frequently observed non-pulmonary organ manifestation in patients with ILD.

## Abbreviations

The following abbreviations are used in this manuscript:

ACMG	American College of Medical Genetics and Genomics
chILD	Childhood interstitial lung diseases
DPLD	Diffuse parenchymal lung diseases
HPO	Human phenotype ontology
ILD	Interstitial lung diseases
MDT	Multidisciplinary team
PAM	(Pulmonary) alveolar microlithiasis
PAP	Pulmonary alveolar proteinosis
PTI/NEHI	Persistent tachypnea of infancy
