# Supplementary material for: Childhood Interstitial Lung Disease—Successful Application of a Stepwise Diagnostic Classification
Source: J Clin Med. 2026 May 21;15(10):3971. doi: 10.3390/jcm15103971 (PMC13207289; doi:10.3390/jcm15103971)
Supplement: Supplementary file 1 [file jcm-15-03971-s001.zip › jcm-4221451-supplementary.pdf]

Supplement

Tab. S1: Demographic data of chILD patients included in secondary data analysis. Data were given as total number and percentages per group or median with first and third quartiles.

	**All patients**	**chILD-EU register patients**	**Munich centre patients**
**Patient's selection**			
Patients with insufficient data, N	206	117	89
Patients with secondary immunodeficiency, N	109	23	86
No material for genetic analysis	302	193	109
**Patients for secondary data analysis, N**	**1693**	**788**	**905**
**Patient's characteristics**			
Age at baseline assessment [years]	1.3 (0.3 to 8.0)	2.4 (0.5 to 9.7)	0.8 (0.2 to 5.4)
Follow-up [years]	7.1 (3.0 to 13.3)	6.1 (3.0 to 8.5)	10.0 (2.9 to 15.8)
Male to female ratio	1 : 0.82	1 : 0.89	1 : 0.77
Age at death/lung transplantation [years]	0.5 (0.1 to 2.7)	0.9 (0.2 to 5.3)	0.4 (0.1 to 1.3)
Deceased/lung transplanted, N (%)	323 (19.1%)	133 (16.9%)	190 (21.0%)
**Geographic Distribution**			
Transferred from European center	1662	780	882
Transferred from non-European center	31	8	23
**Categorization in secondary data analysis**			
ILD related to exposure	421 (24.7%)	123 (15.6%)	298 (32.9%)
ILD with systemic condition	385 (22.7%)	169 (21.4%)	216 (23.9%)
ILD with genetic diagnosis of systemic disease	283 (16.7%)	162 (20.6%)	121 (13.4%)
ILD with genetic diagnosis, affecting lung only	170 (10.0%)	100 (12.7%)	70 (7.7%)
ILD, unknown etiology, without genetic diagnosis	434 (25.6%)	234 (29.7%)	200 (22.1%)

Tab. S2: Most frequently observed non-pulmonary organ manifestation in patients with ILD (N = 1,693). The non-pulmonary organ manifestation was described by standardized human phenotype ontology (HPO) terms, which were linked to major organ groups.

**Major organ groups**	**Top five used HPO terms per organ (N)**
Autoimmune features	HP:0003493: Antinuclear antibody positivity(16); HP:0020050: Anti-granulocyte-macrophage colony stimulating factor antibody positivity (15); HP:0003453: Antineutrophil antibody positivity (9); HP:0025343: Lupus anticoagulant (8); HP:0003613: Antiphospholipid antibody positivity (6)
Lymphatic features	HP:0001744: Splenomegaly (34); HP:0002955: Granulomatosis (20); HP:0002716: Lymphadenopathy (20); HP:0010310: Chylothorax (13); HP:0001789: Hydrops fetalis (8););
Immunodeficiency features	HP:0004313: Decreased circulating antibody level (38); HP:0100806: Sepsis (27); HP:0004432: Agammaglobulinemia (11); HP:0005681: Juvenile rheumatoid arthritis (11); HP:0001974: Leukocytosis (11);
Vascular features	HP:0004890: Elevated pulmonary artery pressure (288); HP:0011726: Persistent fetal circulation (38); HP:0002239: Gastrointestinal hemorrhage (9); HP:0001028: Hemangioma (9); HP:0000421: Epistaxis (8);
Endocrine features	HP:0000821: Hypothyroidism (71); HP:0000836: Hyperthyroidism (10); HP:0000872: Hashimoto thyroiditis (8); HP:0000846: Adrenal insufficiency (7); HP:0000829: Hypoparathyroidism (5)
Syndromic features (incl. head and neck)	HP:0000252: Microcephaly (36); HP:0000365: Hearing impairment (31); HP:0500049: Retinopathy of prematurity (25); HP:0001999: Abnormal facial shape (21); HP:0000407: Sensorineural hearing impairment (11);
Dermal features (incl. hair and nails)	HP:0001217: Clubbing (50); HP:0001047: Atopic dermatitis (25); HP:0000961: Cyanosis (17); HP:0000964: Eczematoid dermatitis (13); HP:4000054: Exanthem (12);
Muscular features	HP:0001252: Hypotonia (117); HP:0000776: Congenital diaphragmatic hernia (18); HP:0000023: Inguinal hernia (17); HP:0012378: Fatigue (9); HP:0001537: Umbilical hernia (9);
Cardiac features	HP:0001631: Atrial septal defect (125); HP:0001643: Patent ductus arteriosus (111); HP:0001655: Patent foramen ovale (89); HP:0001629: Ventricular septal defect (48); HP:0005180: Tricuspid regurgitation (37)
Gastroenterological/ pancreatic features	HP:0002020: Gastroesophageal reflux (85); HP:0002013: Vomiting (36); HP:0002014: Diarrhea (30); HP:0002027: Abdominal pain (24); HP:0002608: Celiac disease (19)
Hepatic (incl. gall bladder) features	HP:0002240: Hepatomegaly (59); HP:0002904: Hyperbilirubinemia (20); HP:0001410: Decreased liver function (16); HP:0001396: Cholestasis (14); HP:0002910: Elevated circulating hepatic transaminase concentration (14)
Hematological features	HP:0001903: Anemia (87); HP:0001873: Thrombocytopenia (51); HP:0001875: Neutropenia (19); HP:0001891: Iron deficiency anemia (18); HP:0001880: Eosinophilia (12);
Neurological features	HP:0001263: Global developmental delay (87); HP:0001250: Seizure (58); HP:0001270: Motor delay (37); HP:0002361: Psychomotor deterioration (30); HP:0012758: Neurodevelopmental delay (29);
Nephrotic/ urogenital features	HP:0000083: Renal insufficiency (23); HP:0000121: Nephrocalcinosis (16); HP:0000093: Proteinuria (14); HP:0000126: Hydronephrosis (13); HP:0012622: Chronic kidney disease (11);
Skeletal features	HP:0002829: Arthralgia (42); HP:0000767: pectus excavatum (42); HP:0002650: Scoliosis (26); HP:0001385: Hip dysplasia (9); HP:0001382: Joint hypermobility (9);
